# Recent advances in bio-integrated electrochemical sensors for neuroengineering

**DOI:** 10.1016/j.fmre.2023.11.012

**Published:** 2023-12-19

**Authors:** Shulin Chen, Tzu-Li Liu, Yizhen Jia, Jinghua Li

**Affiliations:** a Department of Materials Science and Engineering, The Ohio State University, Columbus, OH 43210, USA; b Chronic Brain Injury Program, The Ohio State University, Columbus, OH 43210, USA

**Keywords:** Biochemical sensing, Neuroengineering, Bioimplants, Analytical chemistry, Bio-integrated electronics

## Abstract

Detecting and diagnosing neurological diseases in modern healthcare presents substantial challenges that directly impact patient outcomes. The complex nature of these conditions demands precise and quantitative monitoring of disease-associated biomarkers in a continuous, real-time manner. Current chemical sensing strategies exhibit restricted clinical effectiveness due to labor-intensive laboratory analysis prerequisites, dependence on clinician expertise, and prolonged and recurrent interventions. Bio-integrated electronics for chemical sensing is an emerging, multidisciplinary field enabled by rapid advances in electrical engineering, biosensing, materials science, analytical chemistry, and biomedical engineering. This review presents an overview of recent progress in bio-integrated electrochemical sensors, with an emphasis on their relevance to neuroengineering and neuromodulation. It traverses vital neurological biomarkers and explores bio-recognition elements, sensing strategies, transducer designs, and wireless signal transmission methods. The integration of *in vivo* biochemical sensors is showcased through applications. The review concludes by outlining future trends and advancements in *in vivo* electrochemical sensing, and highlighting ongoing research and technological innovation, which aims to provide inspiring and practical instructions for future research.

## 1 Introduction

Bio-integrated electronics is an emerging field that benefits from the remarkable progress in electrical engineering, biosensing, materials science and biomedical engineering. These technologies are rapidly advancing and becoming increasingly flexible, lightweight, compact, sensitive, biocompatible, and durable [1]. The progresses and breakthroughs are pushing the boundaries of conventional operational modalities, making the resulting electronics highly promising candidates for continuous monitoring of physiological and pathological status during daily activities [2]. This has the potential to reduce the need for prolonged hospitalization and enable individuals to receive timely care and intervention in a more convenient and non-intrusive manner [1].

While remarkable progress has been made in detecting physical and physiological signals, there has been an increasing focus on the detection of biomarkers in bodily fluids in recent years, especially concerning the central nervous system (CNS). Multiple biomarkers in the CNS carry important information for understanding neurological health, diagnosing diseases, and monitoring treatment efficacy: For example, ions are essential for maintaining physiological functions of the nervous system and processing neuronal signaling [3]. Neurotransmitters, such as dopamine (DA) and serotonin (5-HT), serve as essential chemical messengers, with their critical roles in regulating cognitive processes, and any dysregulation can precipitate various neurological disorders [4]. This frontier of research bears significant potential for understanding and diagnosing neurological conditions, as it offers real-time insights into the molecular and chemical changes taking place within the human body. It will also empower users to create personalized databases for customized health management. Given these premises, the demand for technologies that are sensitive, selective, biocompatible, minimally invasive, and spatially resolved has increased. Meeting these needs requires an interdisciplinary approach integrating expertise from multiple traditional fields of technical study.

**Fig. 1:**
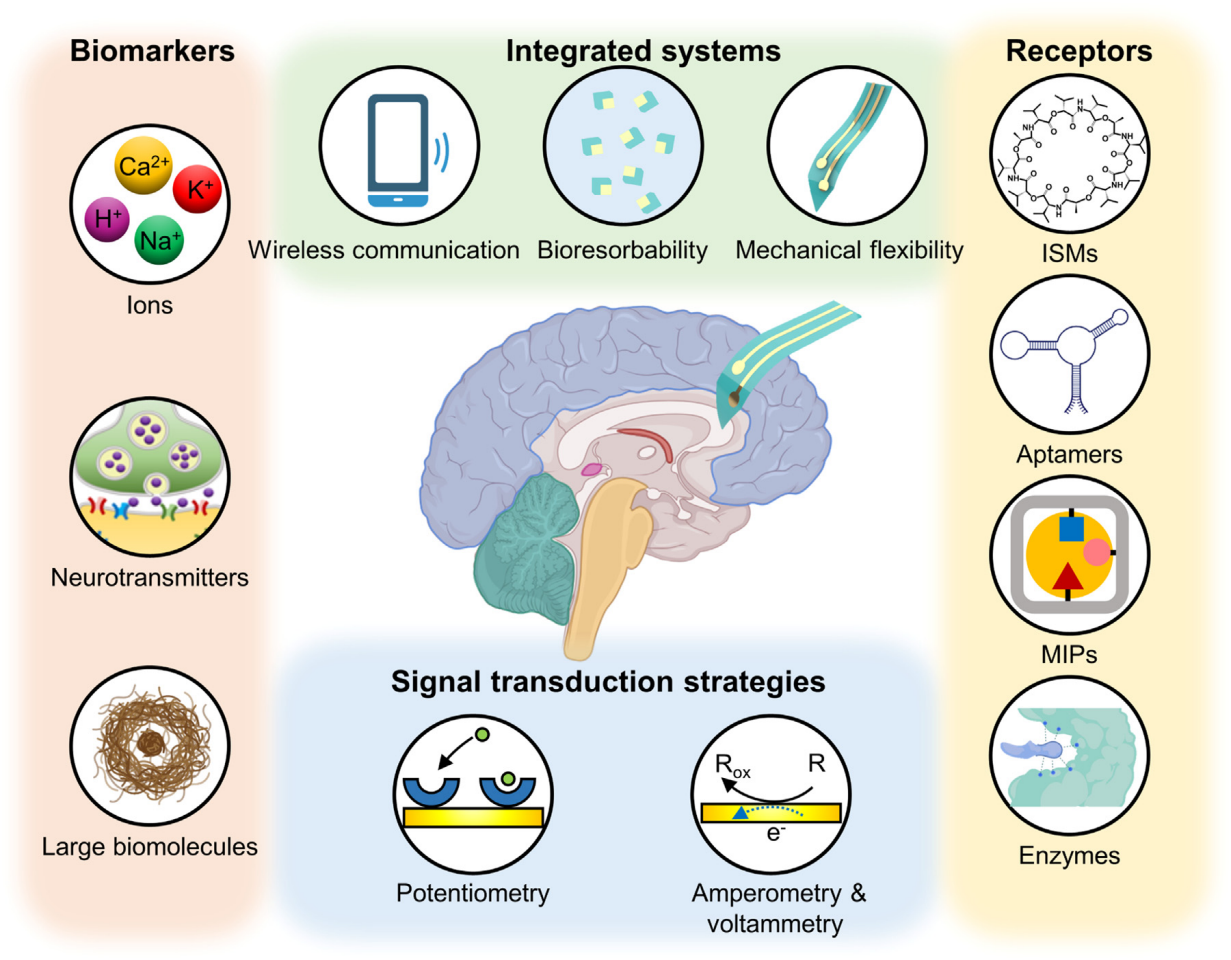
**Schematic illustration of using bio-integrated electronics for detecting biomarkers in neurological diseases.** A variety of biomarkers, such as ions, neurotransmitters, and large biomolecules, are within the scope of detection for disease monitoring, diagnosis and intervention through the integration diverse sensing interfaces, strategies, and wireless communication modules. Part of this image has been generated using Biorender.com.

Herein, we aim to provide a review of the field of bio-integrated electrochemical sensors for the detection of biomarkers, particularly those relevant to neuroengineering. It encompasses a wide range of perspectives, including physiology, analytical chemistry, materials science, and electronics (Fig. 1). We begin this review by providing an overview of significant neurological biomarkers that are of great interest and relevance for biomedical research and translational engineering. Subsequently, we highlight critical aspects of various naturally occurring and artificial bio-recognition elements, which are essential for detecting of neurological biomarkers with high sensitivity and selectivity. We then go on to discuss commonly used sensing strategies and explore the design of transducers, as well as the coupling strategies with the biochemical interface, to achieve the desired sensing performance. Next, we explore the important aspect of wireless signal transmission methods that can be potentially applied for bio-integrated electrochemical sensors. Furthermore, we showcase examples of system integration and diverse applications of bio-integrated neurochemical sensors, focusing on their potential to enable quantitative, continuous monitoring of biomarker levels in living organisms with minimal invasiveness. In the final section, we provide a brief outlook on the future of *in vivo* electrochemical sensing. We discuss emerging trends, challenges, and perspectives on the future of developing and utilizing bio-integrated electronics for neuroscience and neuroengineering.

## 2 Different types of neurological biomarkers

Neurons and neuroglia cells together form a dynamic network in which various chemicals interact continuously. This intricate and ever-changing environment presents opportunities for *in vivo* neurochemical analysis, which offers improved reliability and time resolution compared to conventional *in vitro* analytical methods. Examples of neurochemicals include ions, metabolites, reactive oxygen species, gases, peptides, proteins, and nucleic acids. Monitoring their balance or presence can indicate a variety of conditions such as Alzheimer's disease (AD), Parkinson's disease (PD), multiple sclerosis, traumatic brain injury, peripheral neuropathies, or neurological damages/consequences of COVID-19. Electrochemical sensing also contributes to fundamental insights in analytical neurochemistry due to its exceptional spatial and temporal resolution. In this section, we will provide a concise overview of neurological biomarkers that are of interest for the neuroscience community. Table 1 offers a comprehensive overview of the essential information regarding the biomarkers introduced within this section.

**Table 1:** Overview of different types of biomarkers discussed in this review.

Neurotransmitter	Origin and transport	Normal levels	Related diseases
Various ions		Extracellular fluid (ECF) Na^+^ 135–145 mM *K*^+^ 3.5–5 mM Ca^2+^ 2.2–2.6 mM	Epilepsy, ataxia, migraines, schizophrenia, and Alzheimer's disease
DA “Pleasure neurotransmitter”	Produced in brain, transported by DAT.	CNS: 1–10^6^ nmol/L, Blood: 0–195.76 pmol/L	Parkinson's disease, schizophrenia, depression
Gamma-aminobutyric acid (GABA) “Calming neurotransmitter”	Synthesized from glutamate in the brain catalyzed by glutamate decarboxylase.	Blood: 0.5–3 µmol/L	Epilepsy, mood disorders, autism spectrum disorder (ASD), schizophrenia
Glutamate “Memory neurotransmitter”	Synthesized and stored in specific glutamatergic neurons until released into the synaptic cleft.	Intracellular fluid (ICF) of the brain: 0−10 µmol/L Blood: 40–60 µmol/L	Alzheimer's disease, amyotrophic lateral sclerosis, Huntington's disease, schizophrenia, bipolar disorder, major depressive disorder
5-HT “Mood neurotransmitter”	Produced by neurons localized in the hindbrain's raphe nuclei.	Blood: 0.28–1.14 nmol/L	psychiatric disorders including depression, anxiety, aggression
Norepinephrine (NE) “Alertness neurotransmitter”	Stored in vesicles in nerve terminals, which concentrated it and protected it from metabolism until released following nerve stimulation.	Blood: 0.414–10.049 nmol/L	Major depressive disorder, schizophrenia
Epinephrine “Stress response neurotransmitter”	Synthesized by PNMT from NE, transcription induced by cholinergic stimulation of the adrenal medulla.	Blood: 0–764.3 pmol/L	Pheochromocytoma, anaphylaxis, stress, and anxiety disorders
Acetylcholine (Ach) “Learning neurotransmitter”	Originated from two major places in the brain: 1) basal forebrain and 2) the mesopontine tegmentum area.	ECF of the brain: 0.1–6 nmol/L	Muscle weakness, epilepsy, neurodegenerative diseases, psychiatric diseases, and nicotine addiction
Endorphins “Euphoria neurotransmitter”	Produced primarily by the anterior lobe of the pituitary gland and in POMC cells primarily located in the hypothalamus.	Plasma: 2.0–3.4 pmol/L	Pain relief, stress response, and immune responses (e.g., inflammation). Lower levels associated with aging and Alzheimer's disease.
Neuropeptide Y (NPY)	Synthesized and secreted by neurons in the central and peripheral nervous systems		Alzheimer's disease
Amyloid Beta (Aβ)	Produced in the brain throughout life and accumulates in the cerebral cortex in the elderly	Normal level in CSF > 137.3 pmol/L for individuals > 50 years	Alzheimer's disease

References for neurotransmitter information mentioned: Various ions [3,[5], [6], [7], [8], DA [9,10], GABA [11], Glutamate [12,13], 5-HT [14,15], NE [14,16], Epinephrine [17,18], Ach [19,20], Endorphins [21], NPY [22,23], Aβ [24], [25], [26].

### 2.1 Ions

Ions, such as Na^+^, K^+^, and Ca^2+^, are integral components in maintaining the metabolic homeostasis of the human body. These ions, together with their associated channels, are responsible for generating and transmitting electrical signals between neurons, regulating neuronal excitability, and modulating various physiological processes that govern brain activity, cognition, and behavior [3]. Imbalances in the levels of ions can lead to impairments or deviations from normal health status, giving rise to various conditions such as epilepsy, ataxia, migraines, schizophrenia, and Alzheimer's disease. In traumatic brain injury (TBI), primary injuries occur irreversibly upon impact, damaging blood vessels, axons, nerve cells, and glia. This can lead to delayed secondary injuries, often involving spreading depolarizations (SD) in injured brain tissue. Approximately 40% of TBI patients deteriorate in the days after the initial injury, with SD being a more accurate indicator of secondary injuries and poor patient outcomes. Measuring ion concentrations in brain fluid thus allows early detection of SD which can enable timely intervention to save potentially viable brain tissue [8].

### 2.2 Neurotransmitters

**Fig. 2:**
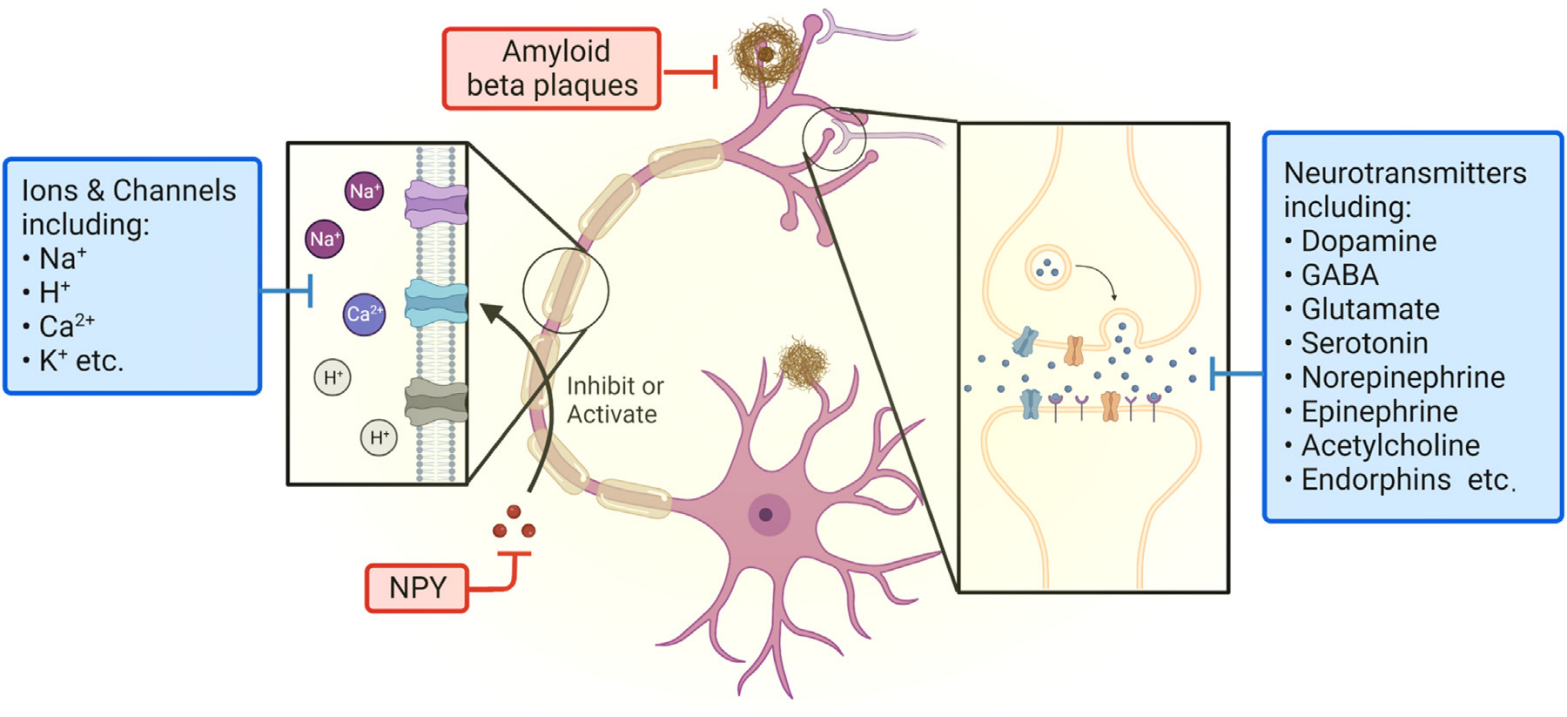
**Important neurotransmitter species found in the CNS discussed in this review.** This image has been generated using Biorender.com.

Neurotransmitters are the primary chemical intermediaries released from neurons to transmit, augment, and modulate signals to other cells. Their functions extend from neuronal activity to endocrine and immune responses, offering the potential to function as clinically pertinent biomarkers for certain disease conditions or in tracking the effectiveness of treatments. In the following part, we will briefly introduce several major neurotransmitters and their unique roles and implications in the functioning of the body (Fig. 2). It is important to highlight that while extensive pioneering studies have investigated biomarker concentrations in the CNS using animal models, there is still a significant need for further exploration of this information in human subjects. The subsequent section provides a summary of the existing literature on this topic.

Glutamate is a primary excitatory neurotransmitter in the mammalian CNS and plays an essential role in regulating neural network signaling transmission through both synaptic and extra-synaptic paths. The concentration of glutamate in the brain intercellular fluid ranges from 0–10 µmol/L, a level considerably lower than that found in blood (40–60 µmol/L) and astrocytes/neurons (10–100 µmol/L) [12]. Glutamate is produced and housed within distinct glutamatergic neurons, poised for release into the synaptic cleft following the receipt of a stimulus [12]. Excessive glutamate release can exert neurotoxic effects, which contribute to neurodegenerative diseases like Alzheimer's, amyotrophic lateral sclerosis, and Huntington's disease [13]. Glutamatergic dysfunction has also been implicated in psychiatric disorders such as schizophrenia, bipolar disorder, and major depressive disorder [13].

The main inhibitory neurotransmitter in the CNS, GABA, acts to counterbalance the effects of glutamate and regulate neuronal excitability. Synthesized from glutamate through the catalysis by glutamate decarboxylase, GABA is found in the systemic circulation of humans at a concentration between 0.5–3 µmol/L [11]. As glutamate and GABA play opposing roles in neuronal signaling, the balance between their signaling is critical for normal brain function. Several neurological and psychiatric disorders, such as epilepsy, anxiety disorders, and depression, are associated with the dysregulation in GABAergic neurotransmission [27].

DA regulates numerous functions such as movement, cognition, mood, and reward. The dopamine transporter (DAT), a protein residing in the plasma membrane, actively shuttles the released transmitter from the extracellular milieu back into the presynaptic neuron, thereby modulating the availability of DA in the brain. Its concentration in CNS typically ranges from 1 to 106 nmol/L, with cerebrospinal fluid displaying a specific value of 0.58 nmol/L [9]. A reduction of 80% or more in DA-producing cells within the substantia nigra has been linked to the manifestation of symptoms in Parkinson's disease. However, the association of DA extends beyond Parkinson's, and its actions surpass the substantia nigra and striatum. DA receptors, present in the reward circuits of the brain, are implicated in phenomena such as the plastic changes observed in drug addiction [10].

5-HT originates from neurons situated in the raphe nuclei of the hindbrain [28]. The 5-HT level in blood typically range from 0.28–1.14 nmol/L [14]. The rate of 5-HT synthesis in the brain varies between males and females, with males exhibiting a production speed of 66–85 pmol/g⋅min and females a rate of 47–55 pmol/g⋅min. [29] Disruptions in serotonergic signaling have been linked to a wide range of neurodevelopmental disorders such as ASD and attention deficit/hyperactivity disorder (ADHD), as well as mental illnesses like schizophrenia and depression [15].

NE is stored in vesicles within nerve terminals and protected from metabolism until released upon nerve stimulation. The normal range of NE in Catecholamine blood test is 0.414–10.049 nmol/L [10]. Despite there is more to explore about α-adrenergic receptors, it is recognized to be in the pathophysiology of major depressive disorder and schizophrenia [16].

Epinephrine is synthesized from NE by phenylethanolamine N-methyltransferase (PNMT) predominantly in the adrenal medulla [17]. In a standard Catecholamine blood test, the normal range for epinephrine is 0–764.3 pmol/L [30]. Epinephrine has been associated with several neurological disorders disorder such as anxiety, dizziness, nervousness, agitation, headache, and exacerbation of PD [18].

ACh is primarily synthesized in two major brain regions: the basal forebrain and the mesopontine tegmentum area. It exhibits relatively low concentrations in the extracellular fluid of the brain, typically between 0.1–6 nmol/L [19]. Alterations in ACh levels or changes in receptor expression and function have been reported in neurodegenerative diseases such as Alzheimer's, Parkinson's, and Huntington's, as well as in psychiatric disorders like schizophrenia [20].

β-endorphins are primarily produced by the anterior lobe of the pituitary gland and pro-opiomelanocortin (POMC) cells mainly located in the hypothalamus [31]. They typically exhibit levels of 2.0–3.4 pmol/L in plasma [21]. As components of the endogenous opioid system and hypothalamic–pituitary–adrenal (HPA) axis, β-endorphins play a significant role in managing pain and moderating immune responses such as inflammation [31]. It is also connected to stress responses as a significant rise can be observed in its serum levels resulting from surgical stress. It has also been reported that older individuals and AD patients exhibit decreased levels of β-endorphins, which could potentially negatively impact neurogeneration and brain plasticity [31].

### 2.3 Large molecules

Large biomolecules such as NPY, Aβ, and tau proteins are particularly pertinent to neurodegenerative diseases, most notably AD [26].

NPY is a 36 amino-acid neuropeptide abundant in the nervous system. It carries out varied functions such as neuroprotection, support for neuronal growth, control of excitotoxicity, calcium balance, and easing neuroinflammation. Both human and animal studies suggest that alterations in NPY levels may be linked to neurodegenerative and neuroimmune conditions [23].

Aβ is instrumental in AD pathology. The disease's key features involve neuritic plaques formed of Aβ peptides and neurofibrillary tangles. The clumping of Aβ peptides instigates a harmful sequence—like hyper-phosphorylation of tau protein and creation of intracellular tau aggregates—resulting in synaptic malfunction, neuronal death, and loss of cognitive function. Both longitudinal and cross-sectional studies imply that changes in Aβ42 levels and tau proteins (both phosphorylated and total) may begin almost ten years prior to the appearance of AD symptoms [26].

AD progresses to irreversible neuronal damage, inducing cognitive decline and loss of motor functions. The ability to accurately measure biomolecules like NPY, Aβ, and tau proteins, however, could significantly aid early detection and potentially allow preemptive interventions. While the damage of AD is irreversible, early detection can still improve disease management and patient quality of life.

## 3 Different types of bio-recognition elements/interfaces

**Fig. 3:**
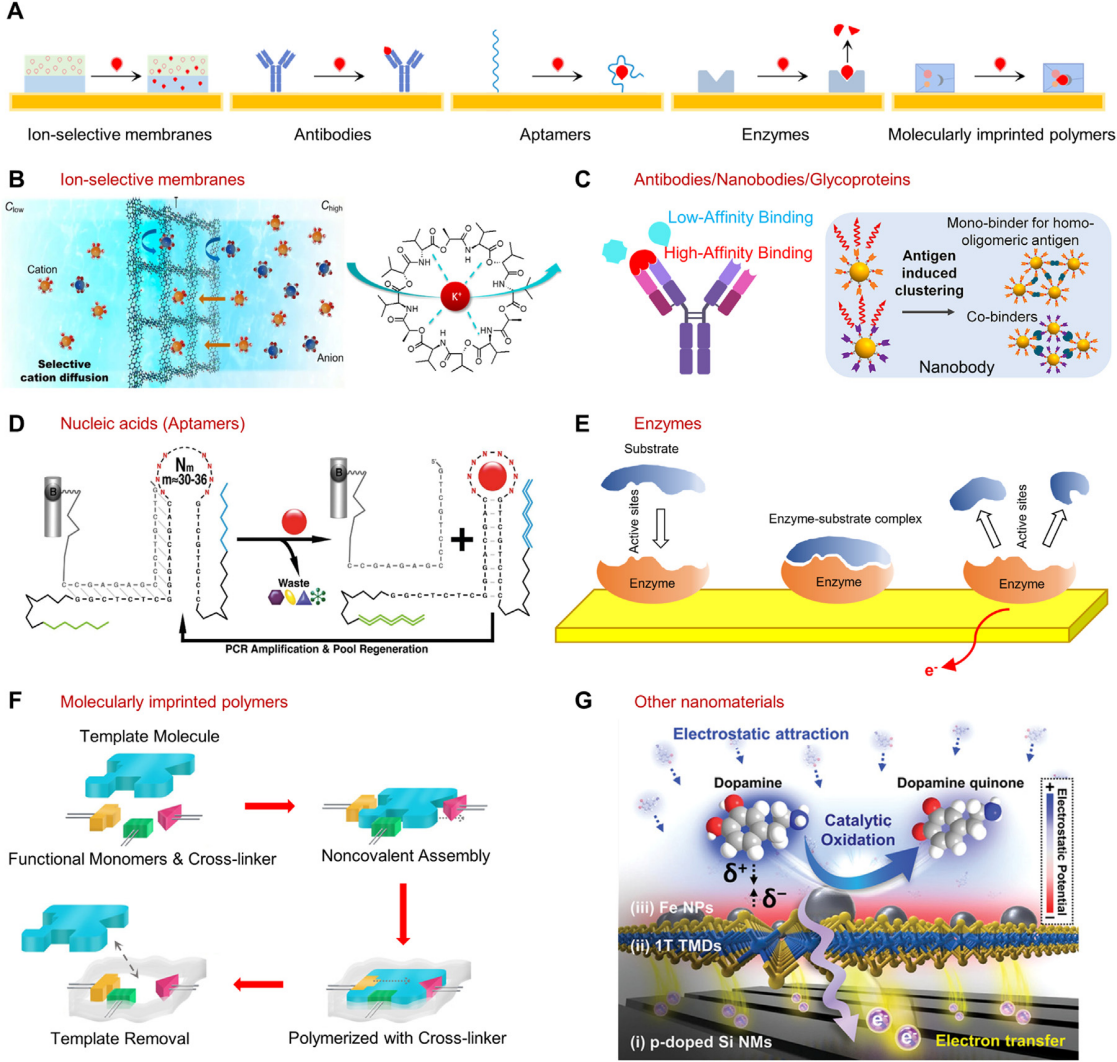
**Representative categories of widely employed recognition elements in biosensing.** (A) Schematic illustration of different kinds of biorecognition elements. (B) Left: selective ion diffusion process across the ion-selective membrane. Reprinted with permission from ref. [32]. Copyright 2022 SpringerLink. Right: structure of valinomycin showing how the cyclopeptide captures and interacts with K^+^. Reprinted with permission from ref. [33]. Copyright 2018 American Chemical Society. (C) Target recognition process based on bioaffinity receptors such as antibodies and nanobodies. Reprinted with permission from ref. [39] Copyright 2022 Elsevier. (D) Process of solution-phase selection of aptamers enabling oligonucleotide receptors for adaptive-loop binding. Reprinted with permission from ref. [40] Copyright 2018 AAAS. (E) Enzymatic catalysis of substrates facilitating charge transfer to underlying electrodes. (F) Fabrication process of MIPs. Reprinted with permission from ref. [49]. Copyright 2018 AAAS. (G) Detecting dopamine without the use of biorecognition elements through monitoring the direct oxidation reaction around a specific electric potential on the electrode surface. Reprinted with permission from ref. [55]. Copyright 2022 Wiley.

Central to the success of biochemical sensing is the effective interaction between biological systems and electronic devices, facilitated by bio-recognition elements. They serve as critical components in the sensing process that enable the analysis of specific biomarkers or biological signals with high precision and accuracy. When choosing bio-recognition elements for specific biosensing applications, several key considerations serve as guiding principles, including sensitivity, selectivity, stability, cost, and compatibility with transducers. This section reviews the diverse landscape of bio-recognition elements and interfaces utilized in biochemical applications. Understanding the principles, capabilities, and limitations is crucial for the development of robust and reliable biochemical sensing platforms in neuroengineering and other related fields. A summary of schematic illustrations showing a variety of bio-recognition elements is presented in Fig. 3A.

### 3.1 Ion selective membranes

Disruptions in the equilibrium of ion levels can give rise to diverse neurological disorders and conditions. To this end, ion-selective membranes (ISMs) allow the selective capture of specific ions for sensing purposes. A common ISM consists of the following essential components: ionophores, lipophilic salts, polymer matrix, and plasticizer. The ionophores are responsible for selectively capturing and binding the target ions. The lipophilic salts help create ion exchange sites within the membranes and minimize interference from counter ions. The polymer matrix provides a structural framework for the membranes and holds the other components together. The plasticizer improves the flexibility and stability of the membrane structure. For example, in the detection of K⁺, a commonly employed combination of components includes valinomycin as the ionophore, sodium tetraphenylborate as the cation exchange sites, polyvinyl chloride (PVC) as the plastic matrix, and dioctyl sebacate (DOS) as the plasticizer (Fig. 3B). The strength and specificity of the interaction between ionophores and ions typically depend on multiple factors such as the size, charge, and coordination chemistry at the binding site. In general, the interaction between ISMs and the target ions is reversible. This characteristic enables repeated measurements and the potential for real-time, continuous monitoring of ion concentrations within an organism such as Na^+^, K^+^ and Ca^2+^ [32,33]. Moreover, the detection of ACh can also be conducted through ACh-selective membranes made of sodium tetrakis[3,5-bis(trifluoromethyl)phenyl]borate (NaTFPB) (ionic exchange sites), calix[4]arene (CX4) (ionophore) and PVC [34].

Despite the advantages of ISMs, it is important to consider potential drawbacks when applying them in practical biosensing applications, especially in the form of bioimplants. The inherent toxicity of ionophores is an important aspect that requires further attention. The leaching of functional components during sensing poses another potential concern, as it introduces foreign ions to living organisms that may disrupt normal physiological processes. The cross-sensitivity between non-target ions can potentially impact the accuracy and reliability of the measurements, especially in complex biological environments where multiple ions co-exist. While recent studies have shown advancements in sensing accuracy through the development of calibration methods that consider specific and non-specific interactions [8,35], further research is required to enhance the performance of *in vivo* applications.

### 3.2 Antibodies/Nanobodies/Glycoproteins

Antibodies represent another important category of widely used bio-recognition elements in biosensing due to their high specificity and affinity for target molecules. They can be designed and engineered against a wide range of targets. As shown in Fig. 3C, the structure of antibodies includes immunoglobulins consisting of two heavy and two light polypeptide chains connected by disulfide bonds. Target analytes can specifically bind to antibodies at the recognition sites through weak chemical interactions including electrostatic interactions, hydrogen bonds, van der Waals forces, and hydrophobic interactions. Biosensors realizing functions based on these antibody-antigen interactions are also referred to as immuno-sensors.

Antibody-based sensors can be categorized as either labeled or label-free immunosensors, depending on if an electrochemical or optical tracer is used. In label-free immunosensors, the direct interaction between an antigen and an antibody generates electrical or optical signals. In labeled ones, immunosensors are often combined with direct signals to detect labeled antigens, with the inclusion of signal labels aiming to enhance analytical signals. Common labels utilized in immunosensors encompass enzymes (such as peroxidase, alkaline phosphatase, and luciferase), fluorescent labels (like fluorescein, rhodamine, and Cyanine5), and redox molecules (including methylene blue (MB), ferrocene, and thionine) [36]. As a commonly used class of biorecognition elements, antibodies have been applied in the detection of neurotransmitters such as GABA [37] and Aβ [38]. Aside from antibodies, nanobodies and glycoproteins can also serve as antigen-binding entities [39]. For example, by linking nanobody to nanoparticles, a colorimetric method has been realized due to localized surface plasmon resonance (LSPR) extinction [39].

While antibodies are highly sensitive, versatile, and widely used, there are certain drawbacks associated with their applications in *in vivo* settings. The strong binding interaction between antibodies and target biomarkers can result in a prolonged association. This may hinder the dissociation of the analytes from the antibody, leading to signal saturation and interfering with subsequent measurements. As a result, using antibody-based biosensors for continuous monitoring without regeneration steps remains challenging. They face issues related to the charge screening effect due to the relatively large dimensions of antibodies compared to the Debye length in the surrounding medium (∼0.7 nm in physiological conditions). Finally, antibodies are susceptible to degradation by proteases and other enzymes in biological fluids. This can result in a reduced lifespan and stability of the sensing interface, limiting the long-term functionality and reliability of the biosensors during *in vivo* applications.

### 3.3 Aptamers

Aptamers have emerged as promising candidates for recognition in biosensing by providing a solution to the challenges associated with the Debye length limitations. Aptamers are synthetic single-stranded functional nucleotides that selectively bind to target analytes. The Systematic Evolution of Ligands by Exponential Enrichment (SELEX) process allows for the *in vitro* selection of aptamers with high affinity and selectivity for specific biomolecules [40]. SELEX starts with the synthesis of a large oligonucleotide library with randomly generated sequences which are then exposed to the target analytes. After filtering, those unbound sequences are removed, and the unique sequences are eluted and amplified. The resulting aptamers have an affinity to a broad spectrum of target including metal ions, small molecules, proteins, and whole cells, [29] which are perfect alternatives to antibodies due to their versatility small size, high detection limit, ease of isolation and modification, and the high stability of nucleic acid ligands for prolonged use in biological environments (Fig. 3D). Previous studies have demonstrated the successful measurement of several neurotransmitters including 5-HT [41], Transforming growth factor-beta 1 (TGF-β1) [42] and NPY [43] using aptamer-based biosensors together with corresponding electrochemical strategies.

Like antibodies, aptamers can also be categorized into labeled and non-labeled formats. After the selective binding to target analytes, aptamers can undergo conformational changes, which are crucial for their functionality and specific recognition properties. The interaction between analytes and aptamers is primarily mediated by non-covalent bonds, including hydrogen bonds, van der Waals interactions, hydrophobic interactions, and electrostatic interactions. The reversibility of these interactions in nature allows for the dissociation of the aptamer-analyte complex under certain conditions. Recent studies have emphasized the potential of allosteric regulation in nucleic acid helices, specifically through the switching between Watson-Crick and non-Watson-Crick interactions, as a powerful tool for controlling the affinity between analytes and aptamers [44]. It provides a mechanism that can potentially enable the aptamers to be reused and regenerated, offering significant cost-effectiveness, and facilitating continuous monitoring *in vivo*.

### 3.4 Enzymes

Enzymes have a long history of being applied as bio-recognition elements due to their remarkable specificity and catalytic activities. They possess highly specific active sites that can accommodate substrates with complementary shapes and chemical properties. These biocatalysts can selectively interact with target analytes and facilitate specific redox chemical reactions involving electron transfer to mediators (first- and second-generation biosensors) or an electrode (third-generation biosensors), resulting in detectable signals through the coupling with transducers (Fig. 3E). Enzymes have been extensively utilized for the measurement of various neurological biomarkers, such as glutamate, ACh, and adrenal [45], [46], [47]. One of the key advantages of using enzymes in biosensing is their non-consumable nature, which enables continuous monitoring of biomarkers without the need for frequent reagents replenishment or replacement of sensing elements. However, the structure of enzymes can be highly sensitive to the environment, leading to challenges in terms of stability and maintaining their effectiveness in physiological conditions. Changes in temperature, pH, or exposure to certain chemicals can disrupt the enzyme structure and cause denaturation or loss of enzymatic activity. As a result, enzyme-based biosensors often exhibit a constrained lifespan, particularly when utilized in *in vivo* applications.

The design of enzyme-based sensors involves several key considerations to ensure their optimal performance due to the variation in characteristics among different enzymes. One important aspect is the selection of an appropriate substrate or platform that provides a conductive and high-surface area surface for immobilizing the enzyme. It is also important to consider the Michaelis constant (*K*_m_) value of the enzyme for the target analyte [48]. If the *K*_m_ value is much higher than the physiologically relevant concentration range of the analyte, the sensor may exhibit reduced sensitivity and limited detection capability. In such cases, a diffusion barrier is necessary to control the flux of biomolecules towards the sensing interface, allowing for better utilization of the catalytic activity of enzymes and extending the linear detection range into the physiological concentration range.

### 3.5 Molecularly imprinted polymers

Molecularly imprinted polymers (MIPs) are synthetic materials designed to mimic the binding properties of natural enzymes and biomolecules by creating specific recognition sites for target analytes. A templating process imprints the target molecules into the polymer matrix. The removal of the template yields binding sites capable of selectively and effectively recognizing the target analytes (Fig. 3F) [49]. MIPs possess notable attributes such as specificity, selectivity, durability to environmental conditions, and cost-effectiveness. The resilience makes them suitable for prolonged and repeated use. In addition, MIPs exhibit a remarkable versatility and universality in their ability to be synthesized for a wide range of target molecules, including amino acids, metabolites, and nutrients [49,50]. There has been a growing interest in the development of MIP-based sensors for the measurement of neurochemicals.

One challenge for MIPs is the complex fabrication process. Furthermore, the extraction of the template molecule subsequent to polymerization can pose a formidable task, which often requires harsh conditions or time-consuming procedures. These factors contribute to the overall difficulty in achieving high reproducibility and scalability in MIP production. Ongoing research aims to address these drawbacks by developing optimized/improved fabrication techniques, expanding their target range, and exploring template removal strategies. Finally, while remarkable progress has been made in the development of MIP-based sensors for neurochemical analysis *in vitro*, their application *in vivo* presents additional challenges that require further efforts.

### 3.6 Other chemical interfaces

In addition to the bio-recognition elements reviewed above, there is a wide range of other biorecognition elements that can be utilized in biochemical sensing applications, including but not limited to peptides, whole cells, viruses, and even whole organisms [51], [52], [53]. Additionally, certain biomarkers produced by the nervous system exhibit inherent electro-active properties and can be detected without the need for specific receptors. The sensing of these chemical species can be further enhanced by integrating nanomaterials such as gold nanoparticles, graphene, and conducting polymers into the sensing platforms. This method is suitable for quantifying electroactive neurotransmitter such as DA, EP, NP, and 5-HT [4]. For example, the detection of DA can be realized using several analytical methods, including cyclic voltammetry (CV), differential pulse voltammetry (DPV), and fast-scan cyclic voltammetry (FSCV) [4,54]. These techniques enable the measurement of the oxidation peak/response waveform of DA, which corresponds to its conversion to dopamine-o-quinone through a two-electron oxidation process (Fig. 3G) [55]. It is also important to note that not all neurotransmitters possess high electrochemical activity. For such biomarkers, alternative detection methods or modifications may be necessary to enable their accurate measurement in electrochemical sensing applications.

In addition to these conventional examples, certain biosensors utilize multiple enzymes or employ indirect detection methods for monitoring analytes. For example, a flexible microsensor can monitor the concentration of GABA using GABase together with glutamate oxidase. One of the main difficulties for enzyme-based GABA detection by GABase is the requirement of a precursor, alpha-ketoglutarate (α-keto), during the reaction. Since α-keto is the product of glutamate oxidation catalyzed by glutamate oxidase, the α-keto required for the GABA reaction to occur is replenished automatically by the glutamate reaction, and the final reporting molecule, H_2_O_2_, gets oxidized on the surface of the sensor [56]. It's important to note that certain analytes can be challenging to detect because they lack electroactivity, are electrostatically neutral, or are present at low concentrations under physiologically concentrations. In such cases, indirect methods can be employed. This approach involves measuring the analyte of interest not through its electrochemical behavior but through a related, secondary reaction or a change in a non-analyte parameter influenced by the presence or concentration of the target analyte. Utilizing redox labels is one of the mostcommonly used methods. One example uses polypyrrole (PPy) MIP as biorecognition element, doped with redox label, hexacyanoferrate (HCF), to measure the concentration of cortisol [57]. Since cortisol and PPy are both electrochemically stable, HCF is necessary to produce redox signal. The binding of cortisol hinders the charge transfer, which results in a decrease in current density. Another example is the introduction of MB to aptamer-base biosensors, which will be further discussed in the next section.

## 4 Strategies for biochemical signal transduction

Having explored the various biorecognition elements utilized in electrochemical sensors, it is also important to review the sensing strategies employed to convert the surface chemistry events into measurable electrical signals. In the field of neuroengineering, accurate and reliable measurements are crucial, especially in the presence of diverse interfering substances and fluctuations in physiological conditions. Achieving this level of precision requires consideration of the design of the signal transducer and the coupling strategy with the biochemical interface. In general, the operational foundation of numerous electrochemical sensors predominantly relies on the utilization of potentiometric, and amperometric and voltammetric detection mechanisms that convert biomarker concentrations into measurable quantities. In this section, we will discuss in detail the representative categories of biochemical sensing strategies and mechanisms that enable the successful translation of neurochemical information into meaningful data.

### 4.1 Potentiometry

Potentiometry is an efficient and attractive analytical technique which can be applied in a broad range of biomarkers. Potentiometric sensors measure the potential difference between a reference electrode and a working electrode: The reference electrode provides a stable reference potential, while the working electrode responds to changes in the analyte concentration or activity. Potentiometric biosensors employ a diverse range of biorecognition elements to enable specific and selective detection of target analytes. Some examples, as mentioned earlier, include ISMs, antibodies, and aptamers.

**Fig. 4:**
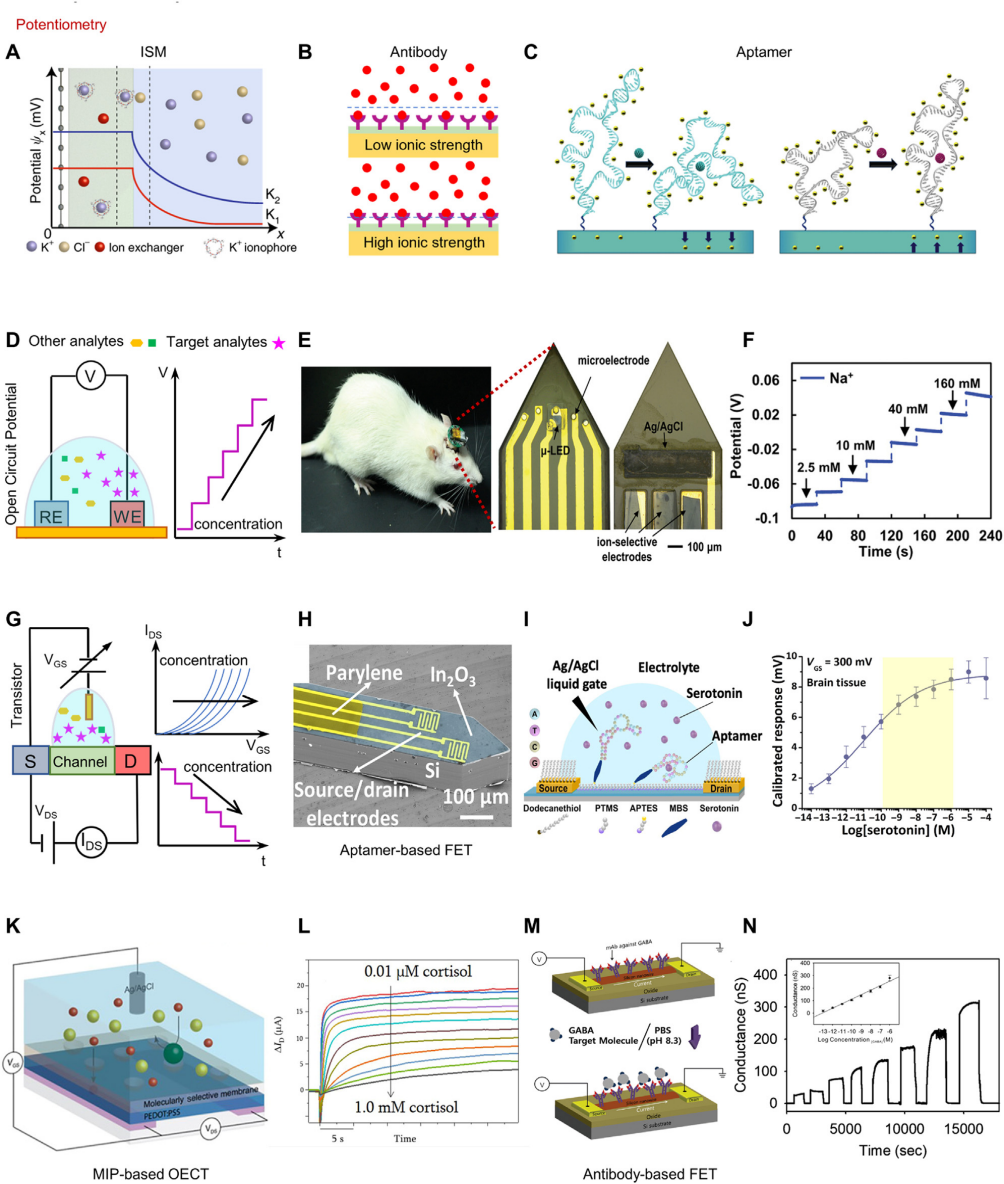
**Potentiometric sensing platforms for detection of biomarkers.** (A) Working principle of ISM-based potentiometric sensors: the selective recognition generates an electrochemical phase boundary potential, transducing the chemical concentration into a voltage signal. Reprinted with permission from ref. [35]. Copyright 2020 SpringerLink. (B) Formation of Debye length (indicated by the dash line) at different ionic strengths and the subsequent effect in the bio-recognition events mediated by antibodies. (C) Mechanism of stem-loop aptamer target-induced reorientations near sensing electrodes and within or near the Debye length. Reprinted with permission from ref. [40]. Copyright 2018 AAAS. (D) Working principle and sensor configurations of a two-electrode system for potentiometry. (E) An implantable flexible device offering opto-electrical stimulation and biophysiological sensing, and (F) Open circuit potential of an ion sensor in (E) responding to varied concentration of Na^+^. Reprinted with permission from ref. [61]. Copyright 2020 Wiley. (G) Working principle and sensor configurations of a solution-gated FET for detection of changes in surface potential. (H) An implantable neuroprobe based on aptamer functionalized FETs for detection of serotonin. (I) Schematic illustration showing the working principle of the probe in (H). (J) Calibrated response (V_th_) of the probe in (H) to serotonin with varying concentrations. Reprinted with permission from ref. [41]. Copyright 2018 AAAS. (K) A An MIP-based OECT for cortisol sensing, and (L) Calibrated response (∆I_D_) of the device to different cortisol. Reprinted with permission from ref. [49]. Copyright 2018 AAAS. (M) A label-free antibody-based transistor, and (N) Change in conductance of the FET in m responding to GABA with varying concentrations. Reprinted with permission from ref. [37]. Copyright 2019 SpringerLink.

For ISM, as the target ion concentration in the electrolytic solution changes, the concentration in the membranes remains constant. A charge separation layer of a few nm thick stays at the interface of sensor surface and electrolyte, and the concentration gradient between the bulk solution and the membranes generates a potential difference across the diffusion layer (Fig. 4A) [35]. The measured potential difference follows the Nernst equation. Antibody-based biosensors utilize the binding between antibodies and target biomarkers to enable the detection and quantification of biomarkers. This binding event can cause changes in the local environment, such as the concentration of ions or charge distribution near the sensing electrode. Consequently, a measurable potential change occurs at the sensor, which is directly related to the concentration of the biomarkers (Fig. 4B). However, antibody-based potentiometric sensors have certain limitations for measurements in high ionic strength solutions [58,59]. This is due to the Debye-screening length (typically < 1 nm under physiological conditions), which represents the distance over which ions in a solution can effectively shield or screen electric fields. The sizes of antibody fragment receptors are often comparable to or larger than this critical length scale. As a result, in high ionic strength solutions, the dense ions surrounding the antibodies can interfere with the accurate detection and measurement of potential changes. Aptamers offer a solution to overcome the limitations posed by the Debye-screening length. Unlike antibodies, aptamers are smaller in size and have more flexibility in their structures. When the target analyte binds to aptamers, it induces conformational changes in the aptamer structure, which can lead to alterations in the distance between the aptamers and the surface of the electrodes. The binding event can either bring the charges closer to or away from the surface of the sensor. This change in the number of charges within the Debye length affects the local ionic environment and the distribution of electric potential near the electrode surface, leading to a measurable voltage signal (Fig. 4C) [40,60].

For potentiometric sensing utilizing the recognition elements mentioned earlier and others, signals can be detected by directly measuring the passive open circuit potential between the modified working electrode and a reference electrode (Fig. 4D). Due to its simple structure, ease of fabrication and compatibility with miniaturization, ISM-based potentiometric sensors represent a prominent category and have found widespread applications in in-vivo neurochemical sensing. Fig. 4E,F show an example of a flexible, multifunctional neural probe equipped with ISM sensors. This device enables the simultaneous stimulation of neurons and the detection of biophysiological signals in multiple encephalic regions. Experiments conducted *in vitro* and *in vivo* have demonstrated the capability in generating optical or electrical stimulation, while sensing 16-channels biopotential and concentration of Ca^2+^, Na^+^, and K^+^ ions in distributed regions [61].

Directly measuring the potential, however, may encounter constraints such as high noise levels and limited sensitivity. One solution is to incorporate amplification techniques utilizing transistors (e.g., field-effect transistors (FETs) and organic electrochemical transistors (OECTs)). In this configuration, the bio-interface serves as the gate electrode, while the potential change is transformed into a corresponding modification in conductance between the source and drain electrodes. Compared to conventional ion-selective electrodes, functionalized transistors offer the advantage of enabling pre-amplification of the local signal, as well as facilitating multiplexing. Fig. 4G shows the schematic illustration of a transistor-based potentiometric sensor and the working principle. As an example, Fig. 4H presents an implantable aptamer–FET neuro-probe for monitoring neurotransmitter levels [41]. The Si substrate-based probes feature nanoscale FETs that incorporate ultrathin In_2_O_3_ films (from 3 to 4 nm) coupled with aptamers for recognition of 5-HT. The subsequent surface charge redistribution is detected by the voltage-gated semiconductor (Fig. 4I). As shown in Fig. 4J, such miniaturized probes enable femtomolar 5-HT detection limits in brain tissue. In addition to 5-HT, aptamer-FETs have shown promise in detecting other biomarkers such as DA, sphingosine-1-phosphate (S1P), and glucose [40,^62^,63]. The versatile combination of recognition elements and signal amplifiers gives rise to a diverse array of biosensors as needed for a wide range of applications. Fig. 4K and L showcase a MIP-based OECT for cortisol sensing [49]. The molecularly selective membrane interacts with cortisol, causing modulation of ion transport to the on poly(3,4-ethylenedioxythiophene) polystyrene sulfonate (PEDOT:PSS) channel and tuning the source-drain current. This system enables the measurement of non-electroactive targets, such as cortisol, without the requirement of an enzyme. Furthermore, Fig. 4M,N illustrates the application of a monoclonal antibody (mAb)-based FET in the detection of GABA [37]. This biosensor enables the rapid and accurate sensing of GABA molecules with a fast response time.

### 4.2 Amperometry and voltammetry

**Fig. 5:**
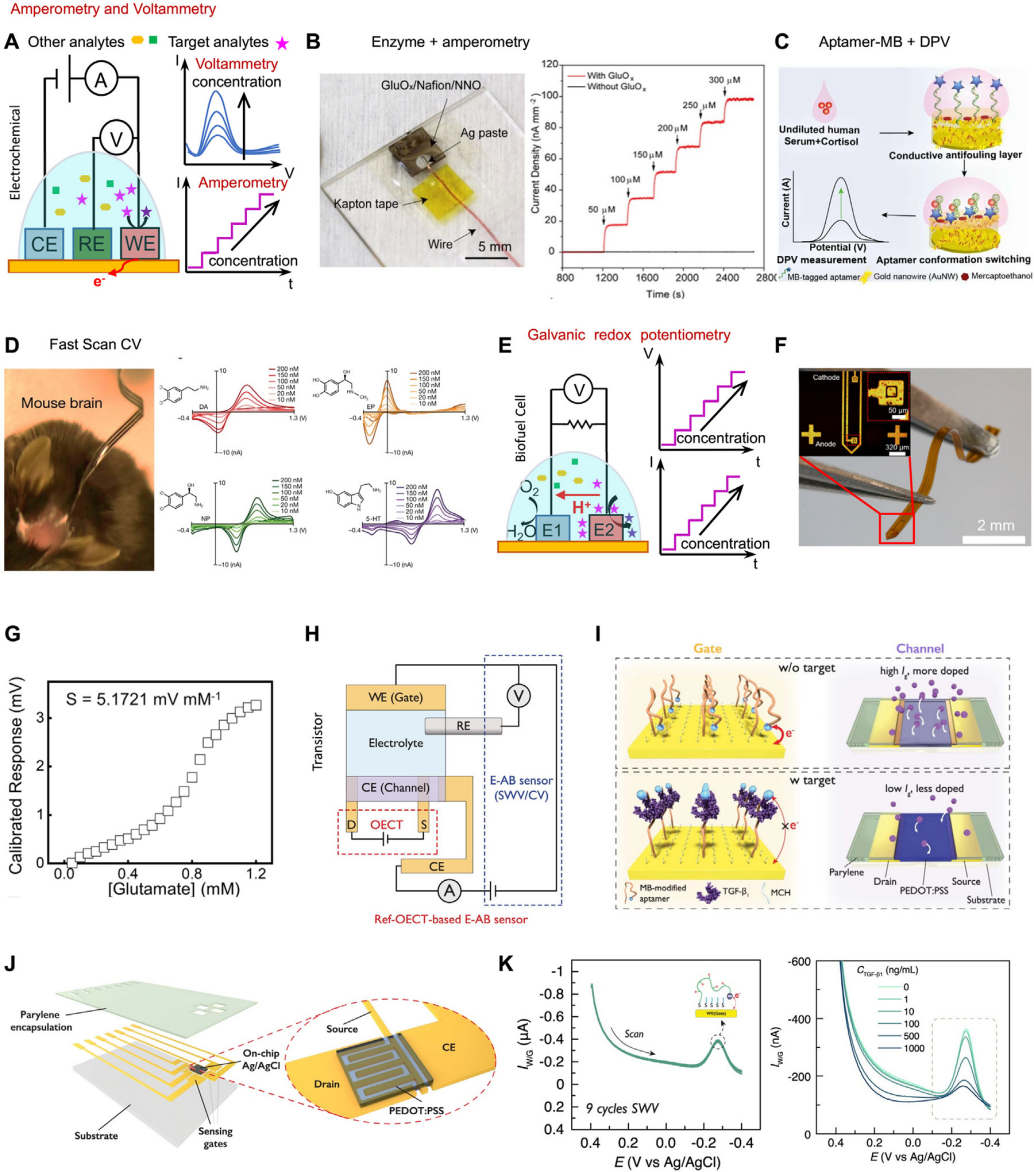
**Amperometric and voltammetric sensing platforms for detection of biomarkers.** (A) Working principle of amperometry and sensor configurations. (B) An in vivo glutamate sensor functionalized with perovskite nickelate-Nafion membrane and glutamate oxidase, and the calibration plot to glutamate with varying concentrations. Reprinted with permission from ref. [45]. Copyright 2020 American Chemical Society. (C) An aptamer-MB-based biosensor detecting cortisol in human serum through DPV. Reprinted with permission from ref. [65]. Copyright 2021 American Chemical Society. (D) An implantable biosensor detecting neurotransmitter including DA, EP, NP and 5-HT using FSCV. Reprinted with permission from ref. [4] Copyright 2022 SpringerLink. (E) Working principle of biofuel cells and the corresponding sensor configurations. (F) An implantable biofuel cell employing a two-electrode configuration (i.e., anode for oxidation and cathode for reduction). Reprinted with permission from ref. [69]. Copyright 2023 Wiley. (G) Measured voltage signal of a biofuel cell sensor responding to glutamate with varying concentrations. Reprinted with permission from ref. [69]. Copyright 2023 Wiley. (H–K) Demonstration of an OECT integrated with an electrochemical cell for amperometric sensing mediated by aptamers, including testing scheme, sensing mechanism, schematic illustration of the device, and SWV sensing results to TGF-β1 with varying concentrations. Reprinted with permission from ref. [42]. Copyright 2023 SpringerLink.

Amperometric and voltammetric biosensors measure the current produced by redox reactions of the target analytes, under a constant (amperometry) or scanning potential (voltammetry). As shown in Fig. 5A, such sensors typically consist of a three-electrode system (i.e., working, counter, and reference electrode) [37]. Enzymatic sensors are widely used in current-based sensing, where specific enzymes catalyze the biomarkers, facilitating charge transfer reactions [45,64]. Fig. 5B demonstrates the use of a perovskite nickelate–Nafion heterostructure for glutamate sensing with a low detection limit of 16 nM and a response time of 1.2 s [45]. Aptamers, when coupled with redox-active molecules, can also serve as promising candidates for ampetrometry/voltammetry. The conformational-switching property of aptamers enables them to control the relative position of redox indicators to the working electrode [65,66]. An example of cortisol sensors based on this design appears in Fig. 5C. A MB-tagged aptamer is immobilized on a gold nanowire nanocomposite to capture cortisol, leading to a change in charge-transfer resistance. This change generates a signal that is proportional to the concentration of cortisol, which is recorded using techniques such as DPV and chronoamperometry [65].

As mentioned earlier, certain amine neurotransmitters like DA, NE, and 5-HT are inherently electro-active molecules. This characteristic eliminates the need for enzymes or other recognition elements for biosensing. To this end, voltammetry can be utilized, which measures the direct oxidation or reduction reaction around a specific redox potential under an applied potential waveform. One example is FSCV, which involves rapidly sweeping the voltage applied to an electrode and measuring resulting current response waveform that is unique to each analyte (Fig. 5D) [4]. FSCV thus allows for the monitoring of the dynamic release/uptake with a temporal resolution. Other examples include DPV and square wave voltammetry (SWV). Compared to CV, DPV and SWV can reduce background charging current and enable precise, sensitive measurements. Due to the absence of specific bioreceptors, however, the selectivity of such methods for detecting electroactive molecules is often limited, as distinct molecules could share a similar redox potential.

In contrast to conventional amperometric/voltametric sensors, an alternative approach in biosensing involves the utilization of a biofuel cell structure, which directly converts the chemical energy of biomarkers into electrical energy for detection (Fig. 5E). The system consists of an anode and a cathode. The anode is modified with specific catalysts to facilitate the oxidation of redox-active fuels (in this context, analytes) present in the biofluid, while the cathode facilitates the reduction of an electron acceptor, typically oxygen. This current can be related to the concentration of the target biomarker present in the biofluid. By incorporating a resistor or a high-impedance voltmeter, it becomes possible to establish a potential that is directly correlated to the concentrations of biomarkers [67,68]. This approach, alternatively known as “galvanic redox potentiometry”, eliminates the necessity for an applied potential, mitigates substantial circuit current, and minimizes notable impact on neuronal firing rates during electrochemical sensing. This is demonstrated by a recent study on a flexible, miniaturized neural probe inspired by the working principle of biofuel cells for ex vivo monitoring of glutamate release from the hippocampal circuit of mice following electrical stimulation (Fig. 5F, G) [69].

Like potentiometry, transistors can also be applied to amperometric/voltametric biosensors for signal amplification. A recent work introduces a class of electrochemical aptamer-based (E-AB) sensors with on-site OECT amplifiers. MB is used as a redox reporter, linked to aptamers specific to analytes. The device monolithically integrates an Au working/sensing electrode, on-chip Ag/AgCl reference electrode, and PEDOT:PSS counter electrode, which also serves as the channel of an OECT. Simultaneously performing testing of the OECT and the electrochemical cell using CV and SWV enables the quantification of biomarkers through amperometry. This device can amplify the current from the electrochemical aptamer-based sensor via the current modulation in the counter electrode/OECT channel through doping and de-doping. The integrated sensor can sense TGF-β1 with 3 to 4 orders of magnitude enhancement in sensitivity compared to that in an electrochemical aptamer-based sensor (Fig. 5H–K) [43]. Table 2 summarizes the detailed parameters of the sensors from the referenced works included in this section, facilitating a comprehensive understanding and comparison.

**Table 2:** **Summary of reported sensor parameters discussed in**Section 4.

Reference number	Sensitivities	Response time	Detection limit	Reversibility	Target analytes
Ref. 35	NH₄⁺: 58.6 mV/decade NO₃⁻: −56.7 mV/decade Na⁺: 49.2 mV/decade K⁺: 45.7 mV/decade Cl⁻: −43.0 mV/decade SO₄²⁻: −22.6 mV/decade HPO₄²⁻: −34.9 mV/decade	–	10 µM	Yes	NH₄⁺ NO₃⁻ Na⁺ K⁺ Cl⁻ SO₄²⁻
Ref. 45	0.327 nA/mm^2^/µM	1.2 s	16 nM	Yes	Glutamate
Ref. 41	–	∼2 s	10 fM	–	5-HT
Ref. 63	–	Order of seconds	10 pM	–	5-HT and DA
Ref. 37	–	–	970 fM	–	GABA
Ref. 65	∼80 nA/mm^2^/nM	< 30 s	0.5 nM	–	Cortisol
Ref. 66	–	–	28 fM	–	Creatine kinase (CK)-MB
Ref. 68	Maximal power density of 31 nW/mm^2^ at 0.25 V and 7.2 nW/mm^2^ at 0.4 V	–	–	Yes	Glucose
Ref. 69	5.1721 mV/mM	∼2 s	0.05 mM	Yes	Glutamate
Ref. 42	290 µA/decade	–	–	–	TGF-β1

## 5 Wireless communication strategies

The emergence of in-vivo biochemical sensors has revolutionized biomedical monitoring, offering unprecedented miniaturization, flexibility, and long-term stability. However, the continued dependence on wired signal transmission has curtailed their potential, particularly during dynamic activities. Historically, the stability of data transmission for these sensors was achieved through hard-wired connections between the devices and an external data reader. However, this approach has inherent drawbacks such as limiting patient mobility, posing infection risks, and potentially causing signal distortion due to physical connection.

To address these challenges, wireless communication technologies have been introduced. This advancement has granted clinicians real-time data monitoring and control capabilities, significantly improving patient management and outcomes. The elimination of physical interconnects reduces infection risks and enhances patients' quality of life by facilitating unrestricted movement. In this section, we briefly evaluate prominent techniques such as Bluetooth Low Energy (BLE), near field communication (NFC), passive inductive coupling, and ultrasonic communication. Each of these methods serves as a viable solution to support *in vivo* biosensors, as they have either been demonstrated in practical applications for biochemical sensing or show promising potential for future use in this field.

When combining electrochemical detection with wireless communication, unique challenges arise, encompassing accuracy, noise interference, data transmission speed, and data integrity. Electrochemical measurements demand exacting precision, necessitating a stable and noise-immune communication channel. Environmental disturbances can introduce interference, potentially masking nuanced electrochemical readings. Systems such as BLE and active NFC come equipped with circuit-level signal amplification and filtering, making them apt for reliable and precise monitoring, even with low-amplitude signals. Rapid electrochemical events underscore the importance of data transmission speed and integrity. Any transmission inaccuracies can result in misinterpretations, especially with vital biochemical data. Digital communication platforms like BLE, active-NFC, and ultrasonic channels integrate data error correction capabilities, reducing transmission errors and accommodating high sampling rates. Power management is also crucial, as electrochemical sensors require consistent voltage and current controls. While systems like BLE, active-NFC, and ultrasonic platforms face challenges in maintaining consistent wireless power delivery, passive-NFC is constrained by its incapacity for amperometry and voltammetry, as these methods require power to facilitate the chemical reactions.

### 5.1 Bluetooth communication

**Fig. 6:**
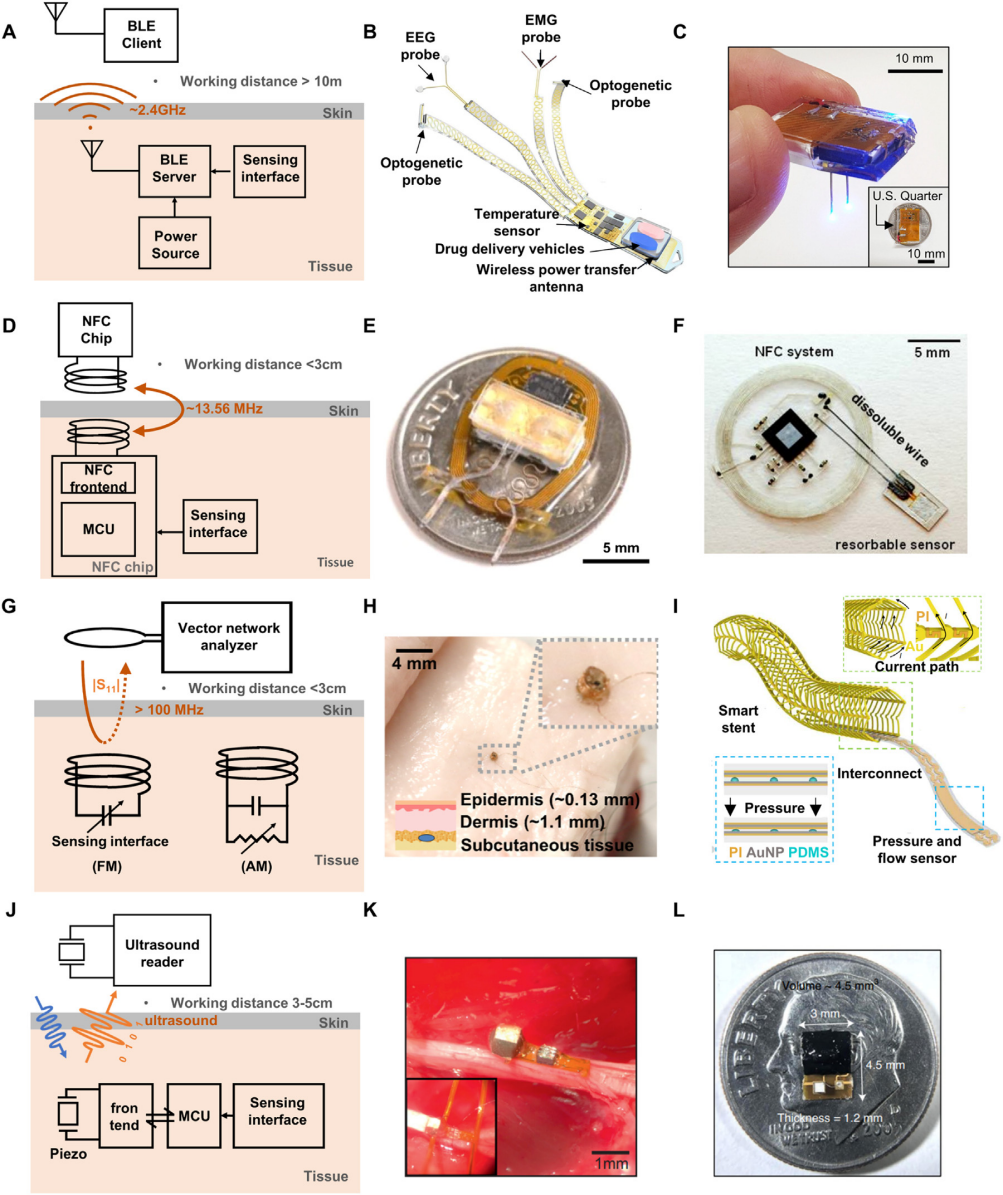
**Overview of wireless communication strategies.** (A) Schematic representation of the BLE communication. (B,C) Examples of representative research employing BLE communication. B, A BLE-enabled implantable multifunctional device capable of monitoring electrophysiological signals (EEG, EMG), drug delivery, and optical stimulation. Reprinted with permission from ref. [70]. Copyright 2023 Springer Nature. C, A BLE-enabled implantable optoelectronic system that can be remotely and selectively controlled via smartphone, with the added capability of wireless recharging. Reprinted with permission from ref. [71]. Copyright 2021 Springer Nature. (D) Schematic representation of NFC communication. (E,F) Examples of representative research utilizing NFC communication. E, An NFC-driven optogenetic device integrated with a microfluidic system for drug delivery. Reprinted with permission from ref. [75]. Copyright 2022 Springer Nature. F, A bioresorbable pressure sensor designed to monitor deep tissue pressure over a predetermined lifespan before resorbing within the body. Reprinted with permission from ref. [74]. Copyright 2016 Springer Nature. (G) Schematic representation of electromagnetic sensing based on a passive inductor–capacitor (LC) resonator, which utilizes either frequency modulation (FM) or amplitude modulation (AM). (H,I) Examples of representative research using passive electromagnetic resonance. H, a tuning circuit inspired LC resonator capable of transmitting electric potential sourced from biochemical signals through frequency modulation. Reprinted with permission from ref. [76]. Copyright 2022 AAAS. I, A cardiac stent integrated with an LC pressure sensor. Reprinted with permission from ref. [78]. Copyright 2022 AAAS. (J) Schematic representation of the ultrasonic communication. (K,L) Examples of representative research using this form of communication. K, A miniaturized nerve recording system that can monitor action potentials generated by peripheral nerves and muscles. Reprinted with permission from ref. [81]. Copyright 2016 Springer Nature 2016 Elsevier. L, An ultrasound-powered implantable luminescence O2 sensor. Reprinted with permission from ref. [82]. Copyright 2021 Springer Nature. neurotransmitter species found in the CNS.

BLE has emerged as a highly suitable wireless communication method for implantable devices due to its minimal energy consumption and robust connectivity [70,71]. As illustrated in Fig. 6A, BLE employs far-field radio waves for signal transmission, thereby extending the data transmission range between implants and corresponding external devices. The robust connectivity ensures reliable data transmission, a feature of high importance for accurate monitoring and control. A recent study has demonstrated a multimodal BLE neural implant that records electroencephalograms, electromyograms, and body temperature, while also facilitating closed-loop neuromodulation via optogenetics and pharmacology as shown in Fig. 6B [70]. Furthermore, BLE-enabled implantable devices allow low energy consumption and less frequent recharging and can be powered by various sources like batteries, radio frequency (RF) power, and triboelectric nanogenerators. Specifically, the wireless recharging capability can enable the elimination of the need for battery replacement (Fig. 6C) [71]. Consequently, a lot of recent research works utilize BLE for building fully integrated bioelectronics in various applications. However, like all active electronics, power efficiency is one such issue that necessitates meticulous management to improve performance and avoid premature battery exhaustion [72]. Signal attenuation in tissue represents another challenge which impacts data transmission quality and range [73]. Consequently, contemporary research focuses on optimizing power consumption and exploring innovative antenna designs to enhance signal propagation, in order to circumvent these obstacles and further advance the application of BLE in bioimplants.

### 5.2 NFC communication and passive inductive coupling

As an alternative to BLE systems, NFC proves its suitability for bioimplants with its compact size and capability to operate without battery or a robust power source [55,^74^,75]. Fig. 6D demonstrates how the coil of an implantable device inductively couples with an external reader. This configuration allows for the harvesting of RF power and facilitates data transmission via the modulation of the RF coupling. The use of RF power from the reader eliminates the requirement for a battery, significantly diminishing the overall device size and weight. This substantially augments the convenience of implantation procedures and minimizes discomforts due to the presence of the device within the body. The technology has been utilized extensively in bioimplant. Among various instances, some recent noteworthy examples include facilitating drug delivery in optogenetic brain implants (Fig. 6E) [75], and monitoring intracranial pressure and temperature (Fig. 6F) [74].

NFC leverages RF power from a reader, transmitting data through inductive coupling, and is categorized into two primary methods: active and passive circuits. Active circuits harvest RF power at 13.56 MHz through inductive coupling, utilizing this energy to power an embedded microcontroller. The microcontroller modulates the inductively coupled antenna, enabling maximum data transmission at a rate of approximately 424 kbit/s. Conversely, passive circuits, comprising solely of passive components such as inductors, capacitors, and resistors, require no power to drive their circuits.

Compared to digital schemes passive resonance circuits working based on inductive coupling are also of interest due to their ability to offer compact, miniaturized, lightweight and flexible designs [76], [77], [78], [79]. As depicted in Fig. 6G, this type of device utilizes inductor-capacitor (LC) resonance where the parameters to be sensed induce variations in a responsive element within the circuit. The resonance frequency [76], [77], [78] or Q factor [79] is thus modulated by the sensing interface, and this response can be quantified to reveal target signals by scanning an inductively coupled reader coil with a vector network analyzer. This method carries advantages such as minimal power consumption, which significantly reduces the size and weight of the device. This reduction, in turn, enhances the biocompatibility of the device and minimizes the invasiveness of the implantation procedure. Current research focuses on enhancing signal stability, system multifunctionality, and seamless integration with medical implants. Fig. 6H showcases a class of battery-free wireless biochemical sensors using a passive resonance circuit inspired by tuning circuits in RF electronics. The interaction between a sensing interface and an LC oscillator, enabled by varactor diodes, translates electric potential changes into modulated capacitance, allowing detection of biomarkers like ions, neurotransmitters, and metabolites [76]. The straightforward circuit design of passive resonators facilitates seamless integration of the resulting devices into electronic systems, as demonstrated by the wireless pressure sensor embedded within a vascular stent (Fig. 6I) [78].

Each method presents its own advantages and challenges. While the active method supports a high data transmission rate, it demands a more complex circuit design, larger coupling antenna, and substantial RF power. The passive method, on the other hand, allows for a significantly smaller device size and lower power requirements, albeit with a limited data transmission rate. Moreover, the working range of passive resonance circuits is typically much shorter than that achievable with other wireless communication methods with integrated digital schemes. This is because the coupling coefficient rapidly decreases with the distance and lateral misalignment between the primary and secondary coils. Current research efforts are focusing on enhancing the communication range by refining the design of the inductive coils and developing advanced signal processing techniques [80].

### 5.3 Ultrasonic communication

Ultrasonic communication is a wireless communication method that utilizes ultrasonic waves for simultaneous power and data transfer between sensing devices and external receivers. Illustrated in Fig. 6J, the implantable device harvests ultrasonic power through a piezoelectric mechanism and utilizes modulated backscatter pulses for transmitting digitized data. Ultrasound offers advantages over RF for wireless communication, including finer spatial resolution, reduced interference, and improved signal propagation through solid materials. Consequently, this approach affords several compelling advantages including high transmission efficiency, minimal signal attenuation in tissues, and compatibility with miniaturized devices. Ultrasonic communication can facilitate real-time monitoring and analysis of physiological signals or neural activity. Fig. 6K illustrates a recording system utilizing this communication principle, showcasing its potential for seamless integration onto peripheral nerves and muscles due to its compact size and enabling the transmission of action potentials via modulated ultrasound [81]. Similarly, the method has the potential of biochemical sensing, In Fig. 6L, an implantable deep-tissue oxygenation sensor has unveiled its potential of integration with biotissue due to the small size and low power consumption [82]. The device concept can readily be expanded to enable *in vivo* wireless sensing of various biological parameters, such as pH and chemical measurements.

Nevertheless, ultrasonic communication has its own challenges. A notable one is signal attenuation in the bone or other dense tissues [82]. Another challenge is the necessity for the ultrasonic wave source to have direct skin contact to ensure effective transmission of wave, given that any air gap or barrier can significantly diminish the signal strength; this can be a limiting factor in certain applications and might cause discomfort or inconvenience to the user. To overcome this obstacle, researchers are exploring advanced techniques such as beamforming and sophisticated signal processing algorithms to enhance signal propagation and alleviate interference effects. Another As such, ultrasonic communication, despite its challenges, holds substantial promise for further optimization and innovation in the realm of implantable devices.

In determining the optimal wireless communication methods for bio-integrated electrochemical sensors, several factors come into play: power management capability, device size/form factors/biocompatibility, data transmission rate, environmental robustness, and signal stability. Active systems like BLE require careful energy regulation, while passive systems like NFC harness energy from external sources, eliminating the need for onboard batteries. The trend towards device miniaturization emphasizes the significance of size and biocompatibility. NFC-supported devices, being more compact, simplify implantation procedures and reduce post-operative complications. This compactness also enhances biocompatibility, improving patient comfort and reducing risks like fibrosis [83,84]. While BLE and NFC both deliver impressive data transmission rates, they each have distinct challenges. BLE boasts an extended range up to 100 m, yet it can encounter signal attenuation from tissue interference, affecting the optimal depth for device implantation [73]. On the other hand, systems utilizing NFC grapple with external electromagnetic interferences and the intricacies of antenna alignment. Ultrasonic methods are efficient through most tissues but can struggle with denser tissue, such as bone, where the high-density tissues can reflect or absorb the ultrasonic waves, diminishing their transmission effectiveness. Despite the inherent challenges of wireless communication, the importance of signal stability in BLE systems cannot be overstated, especially for accurate neural data transmission. The latest version of communication protocol introduces forward error correction (FEC), a feature that enhances the sensitivity of the receiver by incorporating redundant bits [73]. This enhancement improves the signal stability under high signal attenuation. The wireless communication performance specifications are summarized in Table 3, together with the examples of contemporary electrochemical sensors that integrate a variety of wireless communication strategies.

**Table 3:** Summary of different types of wireless communication strategies.

	Far-field communication	Near-field communication	Passive inductive coupling	Ultrasound
Example	BLE	Active NFC	Passive NFC (LRC resonance circuit)	Ultrasound backscattering
Working frequency	∼2.4 GHz	13.56 MHz	> 10 MHz	> 20 kHz
Communication distance in the air	< 100 m	< 10 cm	< 10 mm	N/A; direct skin contact needed
Power consumption	< 10 dBm	0-10 dBm	−10-0 dBm	−4-−9.21 dBm
Max. transmission rate	2 Mbit/s	424 Kbit/s	N/A; analog signal	500 Kbit/s
Limiting factor (size/weight)	Power source (battery, energy harvester)	Coupling antenna	Coupling antenna	Piezo efficiency
Advantages	Long range, allows monitoring during movement, continuous monitoring, supports multichannel operation	Battery-free, supports multichannel operation	Circuit conciseness, battery-free operation, low power consumption rate	Supports deep tissue monitoring, low power consumption rate
Current Challenges	Signal attenuation in tissue; requires high-power source	Requires relatively high-power source	Limited I/O channels; Vector network analyzer (VNA) needed to receive data	Signal attenuation in the dense tissues; Customized ultrasound decoder needed to receive data
Examples	DA (ref. 87)	DA (ref. 55) Catecholamine (ref. 86)	Na^+^ (refs. 76,^85^), Cocaine (ref. 44), K^+^, Ca^2+^, H^+^, 5-HT, Glucose (ref. 76)	Oxygenation (ref. 82)
Ref	[70], [71], [72], [73],^87^	55,[74], [75],^86^	44,76,78,80,76,85	81,82

While the development of wireless electrochemical biosensors is still in relatively early stages compared to their biophysical counterparts, recent years have seen pioneering studies employing the strategies mentioned above, encompassing both wearable and implantable devices. For example, an electronic skin [85] based on LC resonators can monitor the concentration of Na^+^ in sweat continuously. Additionally, a platform enabling the measurement of cocaine concentration utilizes LC resonators to wirelessly transmit the data, which provides the platform capability to function under a variety of scenarios [44]. Some groups have also reported the integration of wireless communication systems into implantable electrochemical biosensors. Examples feature implants equipped with antennas that harvest RF energy using the NFC communication band, aiding in the amperometry to support detection of catecholamine dynamics [86], implantable optoelectrochemical probes wirelessly transmitting DA concentration through BLE [87] and wireless bioresorbable neurochemical systems which can detect DA concentration and transfer signal using NFC [55]. Details will be provided in the next section. As of now, the research community has reported only a limited number of bioelectronics that simultaneously fulfil all three criteria of being implantable, wireless, and electrochemical. For implantable biosensors, a wireless communication system is ultimately essential for the realization of the continuous health monitoring without the need for invasive procedures or frequent medical visits. The concepts showcased in these works can inform future designs in this area. The advances in wireless technologies, such as BLE, NFC and ultrasonic communication, present promising avenues for enhancing the capabilities of electrochemical sensors. The robust connectivity and energy efficiency can be leveraged to improve real-time monitoring in electrochemical sensors. NFC, with its compact design and battery-independent operation, aligns well with the requirements of sensors that necessitate frequent data readings. Passive NFC, characterized by their compactness and low power consumption, offer potential for seamless integration into electrochemical sensing systems. Furthermore, the attributes of ultrasonic communication, including high transmission efficiency and minimal tissue signal attenuation, can be pivotal in the evolution of *in vivo* electrochemical sensors. Harnessing these wireless methods can lead to the development of next-generation electrochemical sensors that are more efficient, user-friendly, and versatile.

## 6 Bio-integrated electrochemical sensors for neuroengineering

Bio-integrated electronics have catalyzed a profound shift in neuroscience through the introduction of *in vivo* electrochemical sensors that monitor brain chemistry. These innovative devices allow scientists to monitor spatially resolved neurochemical dynamics in real-time, providing an in-depth understanding of biological structures and functions in environments mirroring natural physiological conditions. In this section, we illuminate a series of key advancements and strategies that have accelerated progress in this field, with a focus on miniaturization, wireless communication, bioresorbability, and ultra-flexibility, all of which have been essential in facilitating minimally invasive measurements, real-time data transmission, long-term compatibility, and optimal brain contact.

**Fig. 7:**
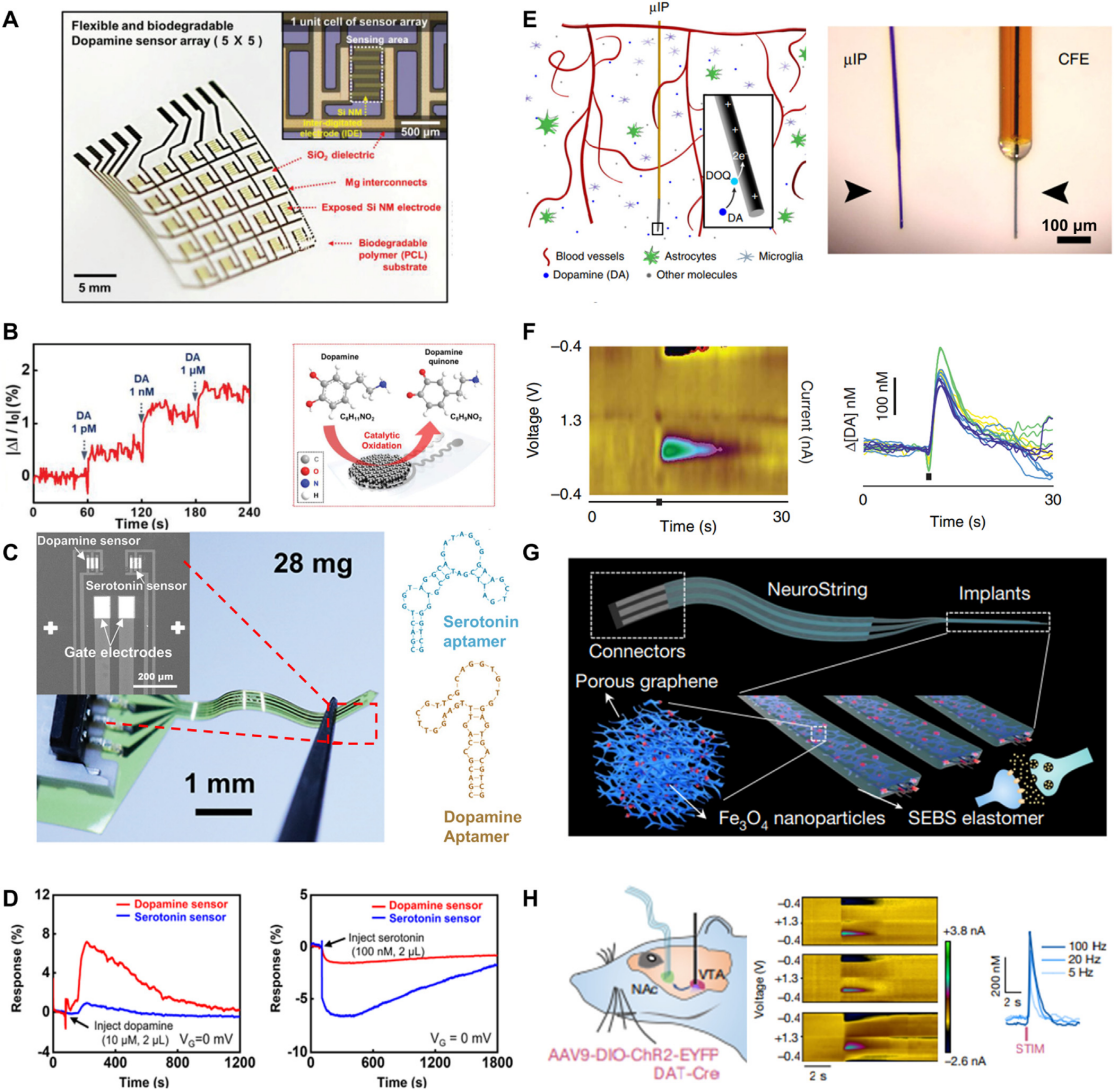
**Recent advances in bio-integrated electrochemical sensors for neurological biomarker detection.** (A-B) Transient silicon nanomembrane-based bioresorbable sensors for dopamine monitoring via amperometry. Reprinted with permission from ref. [88]. Copyright 2018 WILEY. (A) Arrayed device depicted in white light photos. (B) Sensor's calibrated response during stepwise dopamine concentration change. (C,D) Polyimide-based neural probes with G-FETs and aptamers for simultaneous serotonin and dopamine detection. Reprinted with permission from ref. [63]. Copyright 2022 American Chemical Society. (C) White light photos showcasing the probes. (D) Transfer curves of the dual-sensor probe in mouse brain tissue upon dopamine and serotonin injection. (E,F) Semi-invasive microprobe for chronic dopamine monitoring using FSCV. Reprinted with permission from ref. [89]. Copyright 2018 Springer Nature. (E) Schematic and white light photo illustrating the sensor's size. (F) Dopamine measurements via FSCV and Cyclic voltammetry across four days. (G,H) Ultra-flexible sensors for real-time, multichannel monoamine monitoring using FSCV. Reprinted with permission from ref. [4]. Copyright 2022 Springer Nature. (G) Schematic detailing the sensor's properties. (H) Schematic of dopamine sensing in NAc with optogenetic stimulation, accompanied by FSCV and CV responses at three stimulation frequencies.

Ongoing sensing interface research is primarily focused on enhancing the sensitivity and selectivity, as well as its bioresorbability after a predetermined duration. Fig. 7A, B showcase recent works on a class of highly efficient bioresorbable chemical sensing platforms that integrate silicon nanomembranes – serving as a transient interface – and iron nanoparticles (Fe NPs) that catalyze DA oxidation [88]. This pioneering design enables the electrochemical detection of DA through amperometry and fully dissolves in a span of 15 h at room temperature. The transient nature of the materials used in this type of chemical sensing interfaces ensures seamless integration with the tissue, paving the way for their safe dissolution within the body. This innovation opens the door to the development of temporary brain implants capable of monitoring neural transmitters without necessitating a secondary surgery for device removal.

An additional sensing approach involves site-selective functionalization of aptamers onto FETs. Fig. 7C, D illustrate a class of polyimide-based soft neural probes integrated with graphene field-effect transistors (G-FETs) and aptamers, which enable simultaneous monitoring of 5-HT and DA with remarkable response time, sensitivity, and selectivity [63]. Notably, these multiplexed neural probes retain their monitoring capabilities with minimal interference due to the selective grafting of aptamers, thus holding potential for understanding of complex neural processes, interactions, and signaling pathways. Stability studies indicate that the sensors maintain their sensitivity to DA and 5-HT detection after four days of incubation in rat cerebrospinal fluid at room temperature.

One of the significant advancements in this field is the development of cellular-scale probes capable of stable chronic sub-second monitoring of neuronal chemicals such as DA. Fig. 7E,F illustrate a micro-sized sensor with a maximum cross-sectional area of ∼60 µm^2^ that can continuously monitor DA levels by detecting redox currents through FSCV [89]. This sensor is 100 times smaller than conventional carbon fiber electrodes. This minimally invasive design reduces markers of inflammation and tissue damage, significantly contributing to their *in vivo* stability when interfacing with biological tissues. As a result, the sensing platform enables chronic and reliable monitoring of stimulation-induced DA fluctuations for up to a year.

Continuing with a similar rationale aimed at minimizing damage to brain tissues, a class of tissue-like ultra-flexible sensors reported recently offers unique monitoring capabilities for neurotransmitter dynamics (Fig. 7G, H). These sensors, created by patterning a metal-complexed polyimide into an interconnected graphene/nanoparticle network embedded in an elastomer, enable chronic, real-time, multichannel, and multiplexed monitoring of monoamines in the brain through FSCV [4]. Remarkably, these sensors minimize undesired disturbances to peristaltic movements, rendering them particularly useful for investigating neurotransmitter dynamics in the enteric nervous system and the gut. This innovative design also expands opportunities for exploring the influence of neurotransmitters on gut microbes, brain-gut communication, and potential biomolecular sensing in other soft organs.

**Fig. 8:**
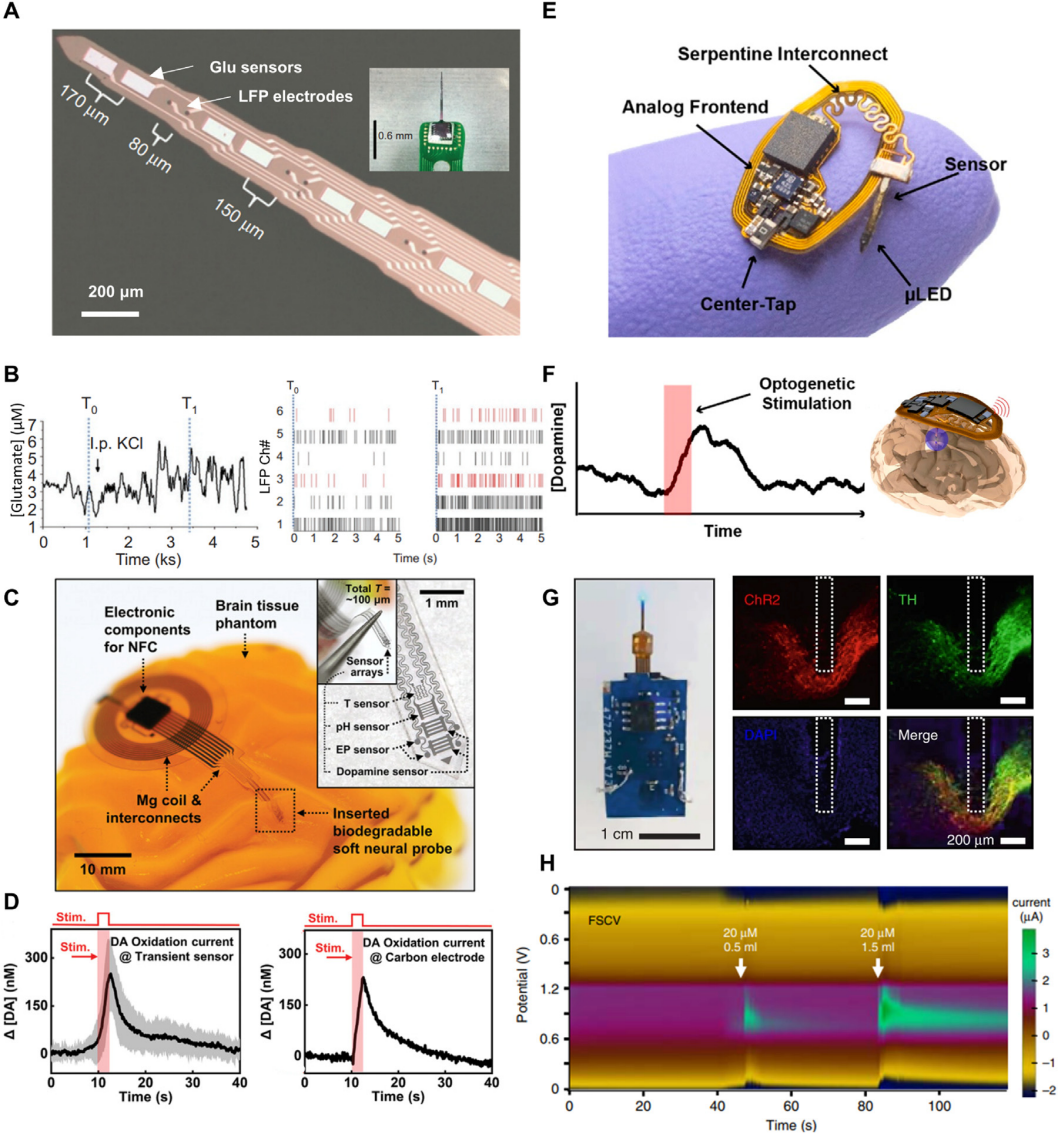
**Recent advances in bio-integrated electrochemical sensors for neurological biomarker detection (continued).** (A,B) Microelectrode array (MEA) for in vivo l-glutamate level recording and electrophysiological signals. Reprinted from ref. [90]. Copyright 2015 Springer Nature. (A) Microscopy details of the sensor shown in white light photos. (B) Glutamate concentration variation post KCl injection (indicated by an arrow) and power spectral densities of the LFPs from multiple channels (C,D) Wireless, bioresorbable silicon sensor transmitting dopamine oxidation current signals via NFC for a set lifespan. Reprinted from ref. [55]. Copyright 2015 WILEY. (C) Sensor details depicted in white light photos. (D) Instantaneous DA responses from the bioresorbable DA components compared to a commercial carbon electrode. (E,F) Battery-free bioimplant with µ-LED and carbon nanotube-based amperometry sensing for multimodal capabilities. Reprinted from ref. [86]. Copyright 2022 American Chemical Society. (E) Sensor details showcased in white light photos. (F) DA transients from the microneedle upon optical stimulation of the nucleus accumbens slices. (G,H) Wireless system for synchronized dopamine level monitoring and optogenetic stimulation, featuring a thin-film diamond for LED heat dissipation. Reprinted from ref. [87]. Copyright 2020. (G) Sensor details in white light photos and immunostaining results of the VTA region expressing ChR2 (top, left), TH (top, right), DAPI (bottom, left), and the merged image (bottom, right. (H) FSCV showcasing varying DA concentration steps in HCl solution at room temperature.

Microelectrode arrays (MEA) represent a classic and effective strategy for achieving precise spatial resolution when monitoring neural transmitter dynamics within the brain. Fig. 8A, B present a microelectrode array specifically designed for simultaneous recording of l-glutamate level and electrophysiological signals *in vivo* [90]. The integrated platinum-based electrochemical (circular, diameter = 15 µm) and electrophysiological (rectangular, 60 × 125 µm^2^) microelectrodes enable dual-mode analytical techniques with exceptional spatiotemporal resolution, which is defined by the microelectrode dimension. This MEA probe demonstrates low impedance and remarkable sensitivity to glutamate, thus facilitates real-time monitoring of extracellular glutamate levels, neuronal spikes, and local field potentials in the striatum of rats. The capacity to detect and analyze dual-mode neuronal signals greatly enhances our comprehension of both the intricate physiology and pathology within the brain *in vivo*.

Another advancement in this field is the integration of biodegradable sensors with wireless communication. This approach provides high-temporal resolution for neurotransmitter monitoring and assures bioresorption within a predefined lifespan, as illustrated in Fig. 8C, D [55]. It showcases a system incorporating a bioresorbable silicon-based electrode embedded with molybdenum/tungsten disulfide (MoS_2_/WS_2_) nanosheets and Fe NPs. These enhancements, especially the nanosheet-iron catalyst hybrids, assist in electron transfer and boost the attraction for positively charged DA, resulting in an improved sensitivity. The system utilizes NFC communication to transfer the signals, maintaining consistent DA tracking for up to 18 days, after which the sensitivity decays due to biodegradation. The NFC transmission facilitates monitoring of DA levels associated with electrical stimulation. The consistency between the wireless system and the gold standard, as demonstrated by researchers comparing the wireless data with commercial carbon-fiber electrodes, further affirms the robustness of this approach. The dissolution of these materials is governed by various factors, including the composition of the materials, the physiological environment, and the presence of specific enzymes or cells that might expedite the degradation process. At a high level, these materials are designed to undergo controlled degradation, breaking down into smaller components through physical and chemical interactions with the bodily tissues and fluids. For example, silicon nanomembranes can turn into flakes in the bodily fluid and dissolute into orthosilicic acid, which can be absorbed by the body [91]. To modulate the degradation rate and functional lifetime of these materials, researchers have pioneered innovative techniques: one approach involves the application of temporary surface coatings that can control the interaction between the material and the physiological environment [92]*.* Another strategy involves the creation of material complexes, where the combination of different materials can influence the overall degradation rate [93]*.* Safety remains at the forefront of these innovations. It's imperative to ensure that as these materials degrade, they neither elicit adverse inflammatory responses nor accumulate as harmful byproducts in organs. Common evaluation strategies involve monitoring for signs of inflammation or tracking the buildup of degradation products in vital organs [91].

In addition to electrophysiology, the combination of optical sources and wireless electrochemical sensors for multimodal operation has introduced compelling and remarkable progress to the field of neuroengineering. One example of such bioimplants, as shown in Fig. 8E,F, eliminates the needs of cumbersome batteries which inhibited the social behavior of animal models [86]. These implants leverage RF power harvesting to enable real-time monitoring of catecholamine dynamics, precise optogenetic stimulation, and in-depth analysis of neural transmitter responses. The miniaturized device incorporates a probe equipped with a microscale light-emitting diode (µ-LED) and a carbon nanotube (CNT)-based amperometry sensing electrode. This setup facilitates real-time measurement of DA fluctuations following optogenetic stimulation, as well as during exposure to opioid and naloxone in freely behaving subjects. The elimination of wires and batteries minimizes interference and boosts the versatility of experimental paradigms. Another example in Fig. 8G, H [87], showcases a BLE-enabled wireless system that integrates µ-LED with an amperometry electrode based on PEDOT:PSS all mounted on a diamond substrate. This combination effectively dissipates the heat generated by the µ-LEDs, preventing potential damage to biotissues. Moreover, the system can analyze the electrochemical properties in a variety of modes, including CV, chronoamperometry, and FSCV. The design also enables synchronization between the optogenetic stimulation and the monitoring of DA levels. These multimodal wireless designs are instrumental in fostering advancements in the field of closed-loop wireless systems. By enabling the real-time tracking of neural transmitter dynamic and optogenetics, these designs offer unique opportunities for responsive neurotherapies.

While this review primarily centers around electrochemical sensors, it is noteworthy to acknowledge that there exists another significant category of biochemical sensors that depend on chemical or biological reactions capable of altering optical detection elements. These alterations encompass changes in light absorbance, such as color shifts, as well as changes in light emission, such as fluorescence or luminescence. For example, a recent study reports the utilization of fiber-optic probes based on engineered cells (FOPECs) enables seamless and real-time detection of neurochemicals in freely moving animals. Leveraging the inherent neurochemical receptors, FOPECs offer unparalleled chemical specificity. Their practical applications include monitoring neurochemical dynamics in real-time across diverse physiological and pathological scenarios [94].

## 7 Conclusion and outlooks

In summary, *in vivo* electrochemical sensing for detecting neurochemicals has garnered considerable interest from various fields, including electrochemistry, materials science, biology, and medicine. The ability to perform real-time measurements of biochemical signals in living organisms has opened new avenues for understanding biological processes and advancing medical research. Electrochemical sensing and analysis offer advantages such as high sensitivity, fast response, and compatibility with miniaturization, making them well-suited for *in vivo* applications. Specifically, the field of bio-integrated electronics is propelled by the remarkable advancements achieved in *in vivo* electrochemical sensing in recent years, providing powerful tools for non-invasive or minimally invasive monitoring of diverse physiological and biochemical parameters. In this review, we have discussed key issues that are essential for the development and application of *in vivo*, bio-integrated electrochemical sensors. The successful integration of bio-integrated electronics relies on the careful selection of various components, considering the specific application scenarios. Several key factors need to be considered, including the sensing interfaces, signal transducers, coupling strategies, electronic modules for signal processing and transmission, and the form factors of the integrated systems. In this context, holistic perspective is essential as it enables researchers to analyze the interdependencies among different components.

Despite the wide research into all the aspects of bio-integrated electrochemical sensors, implantable biosensors still face challenges posed by reactive tissue responses, which diminish the detection of signals and might potentially modify neuron-neuron and neuron-glia activity. The injury caused by device insertion can affect the function of adjacent cells and alter the release and clearance of neurochemicals in the tissue, ultimately leading to a reduction in signal detection over time. To address this challenge, the solution lies in the engineering of device structures and form factors to be flexible and miniaturized, thereby minimizing the risk of injury to brain tissue. Common choices for soft, flexible substrates and ultrathin metal components are often used in combination in this context. Proper encapsulation of the device can further reduce the inflammatory events, glial scarring, and neuronal loss upon probe insertion. Beside this, researchers have explored several methods to modify the surface of implantable biosensor to decrease the inflammatory responses, including (1) coating sensors with hydrophilic materials to prevent the absorption of proinflammatory proteins responsible for recruiting activated glial cells, thereby reducing biofouling; (2) Incorporating bioactive compounds such as cell surface proteins, proinflammatory receptor antagonists, or reactive oxygen species (ROS) scavengers; and (3) using anti-inflammatory and antioxidative substances like dexamethasone [95,96].

Besides, there are also multiple additional aspects that require further improvement: (1) One crucial area is the lifetime and stability of the sensors. Enhancing their durability and long-term performance is essential. While the lifetime of bio-recognition elements is commonly recognized as a key factor in the performance of biosensors, the stability of the electronic transducers is often overlooked. It is important to ensure that all key functional components exhibit sufficient stability to maintain their performance over extended periods of use under physiological conditions. (2) Achieving reversibility is decisive to enable continuous monitoring. Existing regeneration methods often require additional chemical reagents for surface cleaning and re-functionalization of the devices. While these approaches have shown success in restoring sensor functionality, they are not compatible with the development of *in vivo* biosensors. To this end, the design of innovative biorecognition elements to facilitate adaptable and/or reconfigurable performances over time is necessary. (3) The sensitivity and selectivity of biosensors remain to be important aspects requiring careful consideration and improvement. To fully accommodate the requirements of specific applications, viable solutions include optimizing the signal transduction mechanism, enhancing the efficiency of bio-recognition elements, and integrating functional nanomaterials. In a complex environment, undesired crosstalk from non-specific interactions will cause a selectivity challenge. To deal with this issue, one approach is to incorporate different sensors which react to various analytes, especially those with similar structures or sizes that may cause interference signals. The idea is that in a mixed solution, the changes in the measured signals are the total of changes from both targeted and non-targeted interactions. Once data is collected, advanced computational algorithms and machine learning techniques can be harnessed to refine readings, ensuring accuracy by filtering out potential discrepancies or errors [97]. (4) Enhancing the multifunctionality of biochemical sensors and addressing the crosstalk issue to detect multiple analytes simultaneously will provide a more comprehensive understanding of neurochemical interactions in living organisms and patients’ biometric signature profiles. Additionally, integrating electrochemical and simultaneous electrophysiological signals can enhance understanding of neuromodulator systems and neural interactions for clinical applications. However, a compatibility challenge arises from the potential disruption of spontaneous neuronal activity during *in vivo* electrochemical sensing. (5) Other areas for improvement include reducing sample-to-sample and batch-to-batch variation, which can affect measurement consistency, and achieving miniaturized sensing platforms with precise spatial resolution to enable localized and targeted monitoring. Overall, *in vivo* electrochemical sensing is rapidly gaining prominence as a promising approach for neurochemical detection. By tackling these challenges and continuing to innovate, the field of biochemical sensors for neuroengineering holds great potential for advancing our understanding of the brain and improving diagnosis and treatment of neurological disorders. The interdisciplinary nature of *in vivo* electrochemical sensing, which involves the integration of multiple disciplines, creates a fertile ground for continuous basic and applied research.

## Declaration of competing interest

The authors declare that they have no conflicts of interest in this work.
